# Morphological symmetry of the radius and ulna—Can contralateral forearm bones utilize as a reliable template for the opposite side?

**DOI:** 10.1371/journal.pone.0258232

**Published:** 2021-10-06

**Authors:** Eunah Hong, Dai-Soon Kwak, In-Beom Kim

**Affiliations:** 1 Department of Biomedicine & Health Science, College of Medicine, The Catholic University of Korea, Seoul, Republic of Korea; 2 Department of Anatomy / Catholic Institute for Applied Anatomy, College of Medicine, The Catholic University of Korea, Seoul, Republic of Korea; University Hospital Zurich, SWITZERLAND

## Abstract

The most important precondition for correction of the affected forearm using data from the contralateral side is that the left and right bone features must be similar, in order to develop patient-specific instruments (PSIs) and/or utilize computer-assisted orthopedic surgery (CAOS). The forearm has complex anatomical structure, and most people use their dominant hand more than their less dominant hand, sometimes resulting in asymmetry of the upper limbs. The aim of this study is to investigate differences of the bilateral forearm bones through a quantitative comparison of whole bone parameters including length, volume, bowing, and twisting parameters, and regional shape differences of the forearm bones. In total, 132 bilateral 3D radii and ulnae 3D models were obtained from CT images, whole bone parameters and regional shape were analyzed. Statistically significant differences in whole bone parameters were not shown. Regionally, the radius shows asymmetry in the upper section of the central part to the upper section of the distal part. The ulna shows asymmetry in the lower section of the proximal part to the lower section of the central part. Utilizing contralateral side forearm bones to correct the affected side may be feasible despite regional differences in the forearm bones of around 0.5 mm.

## Introduction

Skeletal asymmetry, which has long been a topic of interest among anthropologists, is related to behavioral and genetic factors [1, 2]. Recently, bilateral skeletal symmetry has been widely discussed among orthopedists, who treat patients using patient-specific implants and instruments (PSIs) and/or computer assisted orthopedic surgery (CAOS) in various orthopedic contexts such as the knee, spine, pelvis, femur, and humerus [3–6]. Computed tomography (CT) data of the contralateral unaffected side can serve as a template to determine the true anatomical alignment and shape of the affected side and has several potential advantages. Firstly, the distinct characteristic anatomical features of a patient can be used to more precisely plan surgeries [7]. Secondly, PSIs can be developed [8, 9].

The radius and ulna, which have complex anatomical structures compared to other limb bones, are parallel long bones that have a natural bow [10–13]. The radius and ulna enable the execution of precise movements such as supination and pronation since the radius can pivot around the ulna [14]. Due to the complicated anatomical structure of the forearm bones, malunions can occur in the forearm bones after severe diaphyseal fractures [15–17]. Therefore, appropriate correction of forearm bones fractures is essential, and PSIs and/or CAOS have several advantages [18–20]. According to McDonald et al., using contralateral side data as a template to restore the affected side may have beneficial in accurately defining axis in applying CAOS [20]. In conventional surgery, radiographs and cross-sectional images are utilized during pre-operation procedures to characterize deformities due to of the fractured forearm bones and plan appropriate reduction surgery. However, complicated forearm bone fractures, such as comminuted fractures, it can be difficult to identify the original alignment through radiographs and cross-sectional images. Alternatively, CAOS and/or PSIs, which utilizes 3D images of the contralateral side as a template, might be more effective to restore the exact alignment of the affected side forearm bones.

The most important precondition to correct the affected side forearm bone using data from the contralateral side is that the left and right bone features must be similar [21]. The forearm has complex anatomical structure, and most people use their dominant hand more than their less dominant hand. Therefore, asymmetrical variations of the upper limbs may be present. To apply PSIs and/or CAOS to complex forearm bone fracture repair, the utilization of contralateral side data must be validated. Several studies have assessed the bilateral asymmetry of forearm bones, but their results conflict with each other. Gray et al. reported bilateral similarities of the distal radii [22]. However, Auerbach et al. reported asymmetries in the right upper limb [1]. Vroemen et al. studied about 20 right-handed people and reported that right side bones are generally longer and larger [29]. Bilateral symmetry of the radius and ulna remains a topic of discussion among researchers, and surface shape differences in bilateral radius and ulna are not fully understood with regard to utilizing PSIs and/or CAOS. We expected the external shape of the left and right forearm bones to exhibit differences. The aim of this study is to investigate anthropometric differences of the bilateral radius and ulna through quantitative comparison of whole bone parameters (length, volume, bowing, and twisting) and regional shape differences of the radius and ulna.

## Materials & methods

### Ethics and cadaver CT data

This study was conducted in compliance with the law about Act on Dissection and Preservation of Corpses of the Republic of Korea (act number: 14885) and was approved by the Institutional Review Board of College of Medicine, the Catholic University of Korea (No.: MC20EAS10103). All methods were performed in accordance with the relevant guidelines and regulations. The CT images used in this study were selected from the Catholic Digital Human Library, which was constructed by CT scans of the cadaver with the approval of the same committee (No.: CUMC10U161). The written informed consent for the use of the cadaver and the consent for the use of future research on the related materials were carried out by all donors or authorized representatives.

### Materials

#### CT data selection

We selected 132 bilateral radii and ulnae from 61 females and 71 males collected between April 2004 to December 2020 for this research. We excluded CT data indicating fractures or deformities of the forearm bones. The average age of females was 62 years, and of males 60 years. The average height of females was 156.09 164.14 cm, and of males 164.14 cm. CT data were scanned using a customized protocol for scanning cadavers that was established by the Catholic Digital Human Library (November 2003 to present) [23–26]. Slide thickness of the reconstructed image was in the Z-direction, and the pixel sizes of our scanned data were 0.33 to 0.42 mm. X-ray generating power was 224 mA, and 120 kV. The CT images had less than 0.75 mm thickness, and 0.33 mm to 0.42 mm pixel dimensions. CT images that were scanned with a calibration scale (plastic ball diameter = 2.25 inches) were adjusted to yield real size data.

### Methods

#### 3D modeling processing

CT data were imported into 3D modeling software program Mimics (Version 22.0, Materialise, Belgium) to check the quality of CT images and 3D models. To compare differences between bilateral forearm bone features, solid 3D models were created. Each 3D model surface was formed with triangle mesh. Smaller triangles were used to represent more complex features and achieve more accurate analysis. Therefore, we refined the maximum edge length of the 3D model’s triangle mesh to 0.2 mm.

#### 3D model alignment and registration

**Fig 1:**
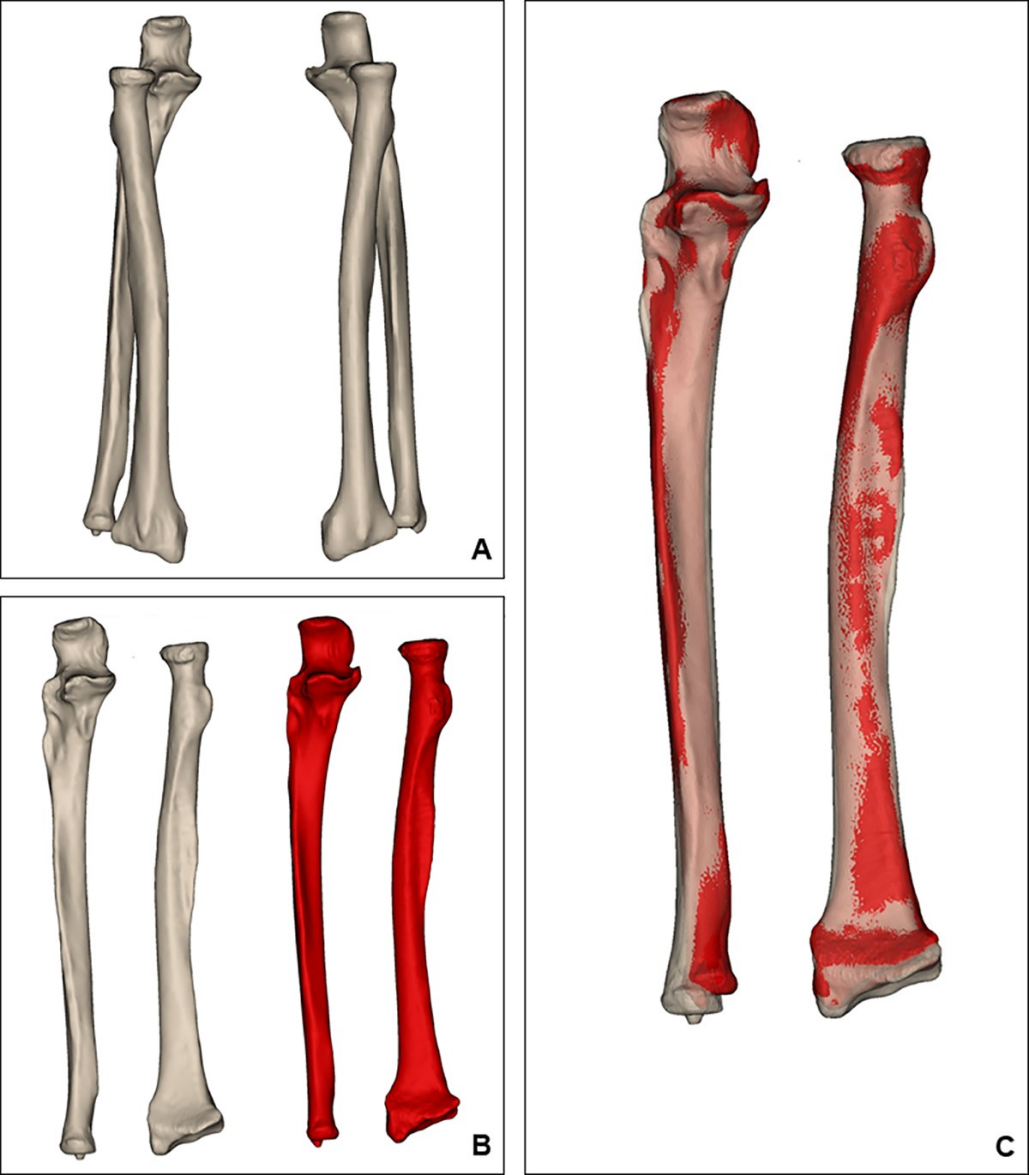
3D model alignment and model registration process. **(A)** We imported 3D models of the radius and ulna. **(B)** The left forearm bones were geometrically reflected, and all forearm bones were aligned to an anatomical coordination system. **(C)** The radii and ulnae were overlapped.

The bilateral radius and ulna data were aligned to an anatomical coordination system. After forearm bone alignment, the left forearm bones were geometrically mirrored. mirrored left forearm bones and non-mirrored right forearm bones were overlapped to compare length, volume, and shape (Fig 1). The overlap registration method that we used is the iterative closest point (ICP) algorithm, which is considered an appropriate method to coordinate two free-form surfaces [27].

#### Measurements

**Fig 2:**
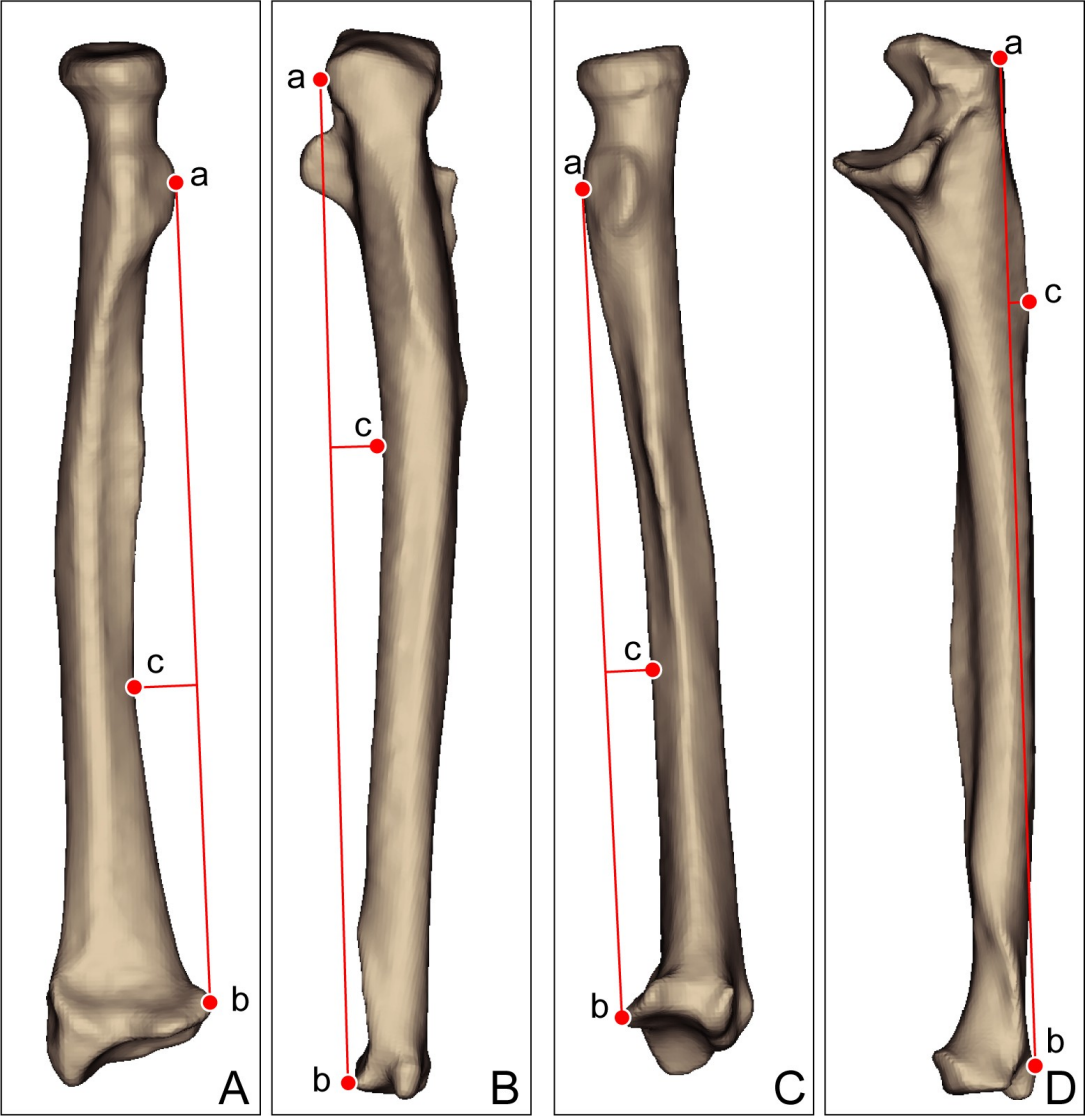
Measurement of forearm bone bowing. The a-b line is defined as ‘bow length’, the location of point c on the a-b line is defined as ‘bow location’, and the distance from line a-b to point c is defined as ‘bow depth’. (A) Coronal bowing measurement of the radius. a, the most ulnar aspect of the bicipital tubercle; b, the most ulnar aspect of the articular surface; c, the farthest radial concavity point from the a-b line. (B) Coronal bowing measurement of the ulna. a, the most prominent ulnar point on the olecranon process; b, the most ulnar aspect of the ulnar head; c, the farthest ulnar concavity point from the a-b line. (C) Sagittal bowing measurement of the radius. a, the most bolar aspect of the bicipital tubercle; b, the most bolar aspect of the articular surface; c, the farthest radial concavity point from the a-b line. (D) Sagittal bowing measurement of the ulna. a, the tip of the olecranon; b, the most dorsal aspect of the ulnar styloid process; c, the farthest ulnar concavity point from the a-b line.

Aligned and registered bilateral forearm bones were imported to the house code using a scientific programming language (Matlab, R2019a, Mathworks, USA) to compare differences of left and right bones. The programmed language was used to measure length, volume, bowing, twisting and distance from centroids to cortical bone outlines of each cross-section. To measure length and volume, the most proximal point and the most distal point were calculated. Coronal and sagittal bowing measurements were performed using the method used by Weber et al. [28] (**Fig 2)**.

**Fig 3:**
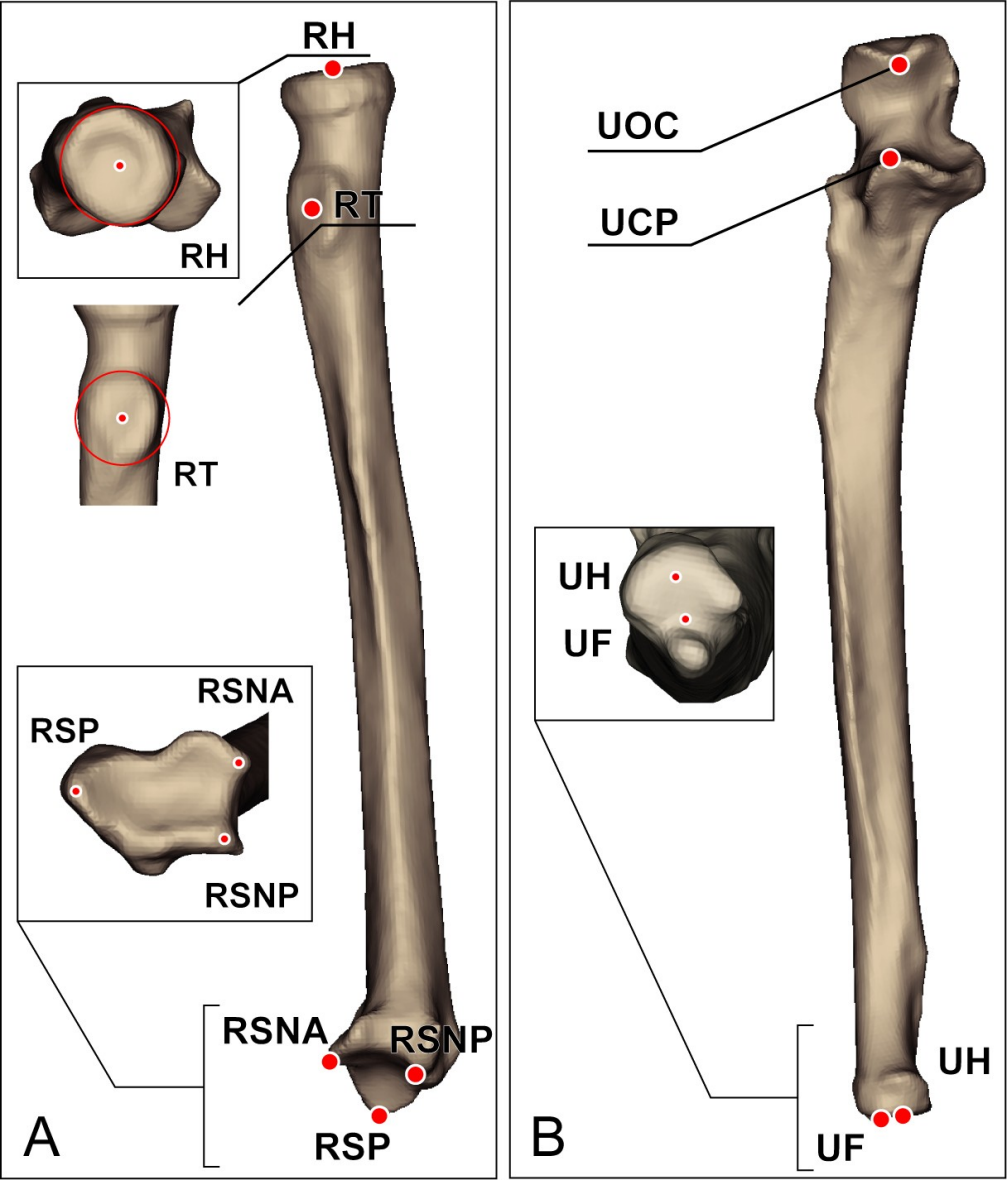
Landmark points on the forearm bones. **(A) RH**, the radial head; **RT**, the radial tuberosity; **RSP**, the radial styloid process; **RUNA**, ulnar notch anterior lip of the radius; **RUNP**, ulnar notch posterior lip of the radius. **(B) UOC**, olecranon tip of the ulna; **UCP**, coronoid process of the ulna; **UF**, the ulnar fovea; **UH**, the ulnar head.

Twist angle was investigated using Daneshvar et al.’s method [29]. Radial twisting was calculated using the angle between the line of the proximal radius that connects the radial head (RH) and the center of the radial tubercle (RT), and the line of the distal radius that connects the radial styloid process (RSP) and the midpoint of the ulnar notch anterior lip (RSNA) and the ulnar notch posterior lip (RSNP) of the axial plane. To measure ulnar twisting angle, the angle between the proximal ulnar line that connects the olecranon tip (UOC) and the coronoid process (UCP), and the distal ulnar line that connects the ulnar fovea (UF) and the center of the ulnar head (UH) was calculated (Fig 3).

**Fig 4:**
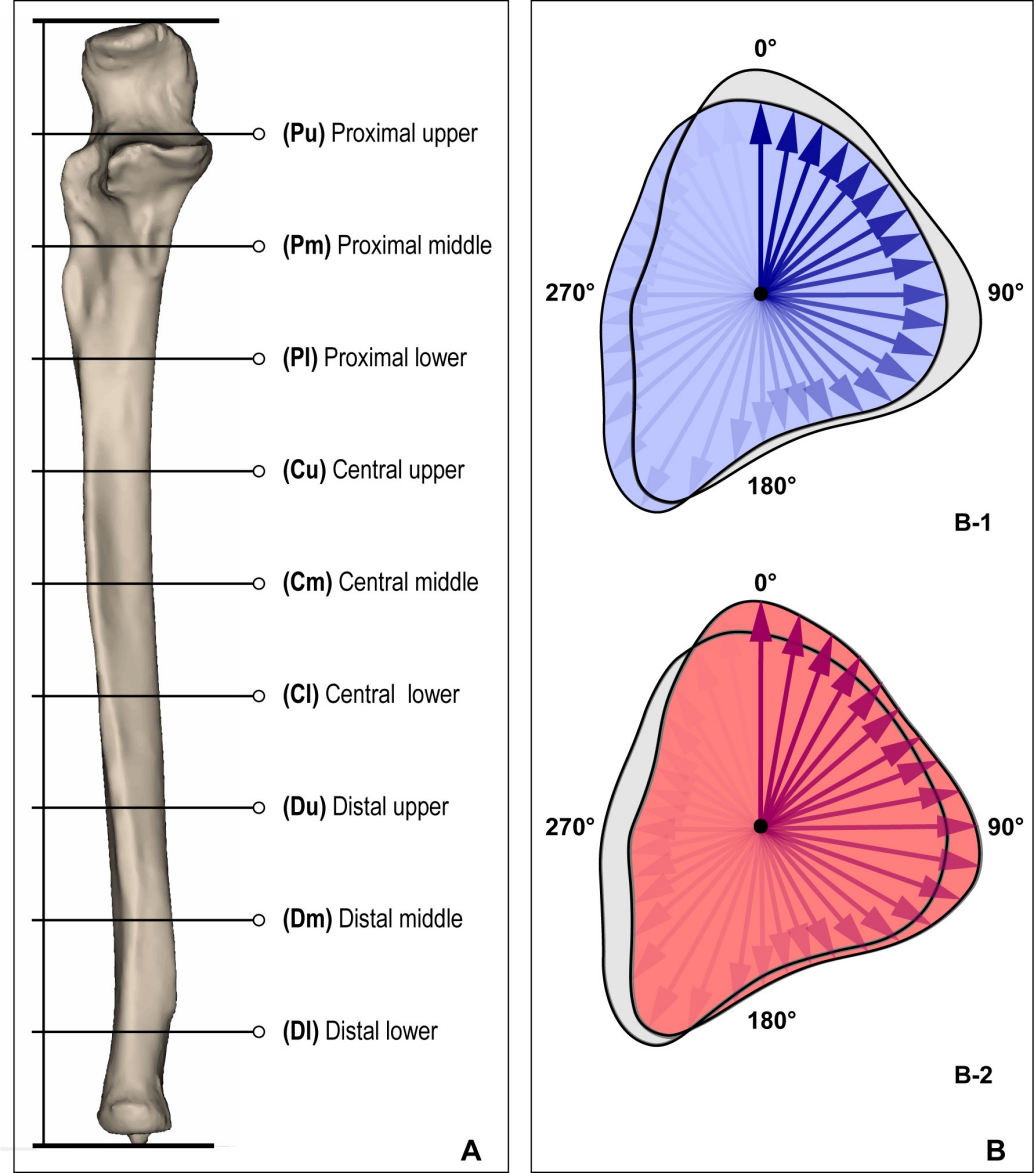
Measurement method of regional shape differences. **(A)** The 3D bone model was divided into 10 equal parts to obtain 9 cross-sectional images. **(B)** Cross-sectional image of overlapped 3D bone models of the original and reflected sides. **(B-1)** Measurement of distance from the right-side centroid to original (right) bone cortical outline. **(B-2)** Measurement of distance from the right-side centroid to reflected bone cortical outline. To characterize the asymmetry of the left and right bones, both bones were measured using centroids from the original side.

To measure distance from centroid to cortical bone outline of each cross-section, 3D models were divided into 10 equal parts. In each part, the centroid of the sectional image was calculated and then the distance from the centroid to the outer surface along with radial direction were measured. Distances of centroids to cortical bone outline measurements were determined 36 times per 10° for 9 sectional images of the 3D bone model of the original (right) side. Then, the contralateral bone (left) was reflected and measured in the same way as the original side, but with the reflected side bone distance from centroid to cortical bone outline measured using the original side centroid (Fig 4).

#### Statistical analysis and data reproducibility

All data were tested for normality of distribution using the Shapiro-Wilk test, and independent t-tests were performed. The level of significance was set at *P* < 0.05. We examined two-tailed t-tests to certify the equivalence of the differences in the bilateral radius and ulna, and the level of significance was 0.05. All length and volume differences were compared between sexes and sides using independent T-tests. Shape differences of the radius and ulna were also compared between sides utilizing independent T-tests. To assess the reliability of alignment, registration and landmark point creation methods, we randomly selected 14 cases from the original data and randomly re-aligned the selected bones using the same registration method. Landmark points were re-created on the selected bones. Length, twisting, bending and regional measurement data were compared with ICC tests.

## Results

### Whole bone parameter analysis of the radius and ulna

We describe significant shape differences of the forearm bones in the S1 File.

#### A. Length and volume

Table 1 show the results of paired T-tests comparing length and volume. Mean lengths of the radius and ulna were 210.01 ± 10.92 mm and 227.10 ± 10.98 mm for females, respectively, and 228.60 ± 11.22 mm and 245.29 ± 11.04 mm for males, respectively. The average length of the radius in males was 18.59 mm longer than in females (p < 0.01). The average length of the male ulna was 18.19 mm longer than in females (p < 0.01). The average volumes of the radius and ulna were 31599.71 ± 5458.02 mm^3^ and 36845.46 ± 5677.71 mm^3^ for females, respectively, and 45880.28 ± 6976.98 mm^3^ and 53146.00 ± 7101.96 mm^3^ for males, respectively (p < 0.01). Paired T-tests revealed that there were statistical significances of length and volume differences of the both forearm bones between the female and male.

**Table 1:** Length and volume of the radius and ulna.

		Female		Male		Combined		P
		Mean	SD	Mean	SD	Mean	SD	
Radius	Length (mm)	210.65	11.50	228.59	11.19	220.57	14.41	<0.01
	Volume (cm^3^)	31.95	5.99	45.99	6.83	39.713	9.52	<0.01
Ulna	Length (mm)	227.80	11.73	245.23	10.92	237.44	14.22	<0.01
	Volume (cm^3^)	37.30	6.49	53.23	6.89	46.11	10.39	<0.01

Side differences of length and volume and paired T-tests are presented in Table 2. There were no significant differences in right and left female radius measurements. The average length of the right radius was 1.46 mm longer than the left (p = 0.48). The average right-side female ulna was 1.85 mm longer than the left (p = 0.37). The average right radius and ulna were longer than the left in males by 1.59 mm and 1.49 mm, respectively. There were no significant differences between bilateral forearm bone measurements in either sex (0.38 ≤ p ≤ 0.41).

**Table 2:** Differences of length and volume between sides.

		Female					Male				
		Right		Left		P	Right		Left		P
		Mean	SD	Mean	SD		Mean	SD	Mean	SD	
Radius	Length (mm)	211.35	11.31	209.95	11.75	0.48	229.41	11.04	227.76	11.34	0.38
	Volume (cm^3^)	32.15	6.09	31.74	5.93	0.67	46.56	6.99	45.42	6.67	0.36
Ulna	Length (mm)	228.71	11.57	226.88	11.93	0.56	245.97	11.27	244.48	10.57	0.89
	Volume (cm^3^)	37.47	6.31	37.12	6.71	0.85	53.66	6.91	52.80	7.02	0.70

#### B. Bowing

The length of right coronal bowing of females was 171.38 ± 10.55 mm and the left was 170.01 ± 10.63 mm (P = 0.510). The location of bowing in the coronal plane was 96.67 ± 13.84 mm in the right female radius, and the left was 96.67 ± 9.49 mm (P = 0.900). The coronal bowing depth of the right female radius was 10.45 ± 2.08 mm, and 10.38 ± 1.77 mm in the left (P = 0.854). In males, the length of the right radial bowing was 184.83 ± 9.77 mm, and the left was 183.29 ± 9.87 mm (P = 0.359). The coronal bowing location of the right male radius was 104.43 ± 9.65 mm, and that of the left was 102.56 ± 9.31 mm (P = 0.248). The coronal bowing depth of the right male radius was 11.95 ± 1.85 mm, and that of the left was 12.15 ± 1.90 mm (P = 0.533).

The measured length of sagittal bowing in females was 171.92 ± 10.39 mm on the right side, and 170.92 ± 10.45 mm on the left (P = 0.623). Sagittal bowing in females was located at 111.67 ± 23.08 mm for the right, and 104.81 ± 22.26 mm for the left (P = 0.126). The bowing location in the sagittal plane of females was 7.76 ± 2.93 mm for the right, and 7.93 ± 3.01 mm for the left (P = 0.770). In males, right sagittal bowing was located at 183.87 ± 10.76 mm, and left at 182.53 ± 9.44 mm (P = 0.439). The bowing location in the sagittal plane of male was located in 119.02 ± 25.96 mm for the right, and the left was 118.91 ± 24.52 mm (P = 0.980). Sagittal depth was 9.99 ± 3.07 mm and 10.02 ± 3.40 mm for the right and left, respectively (P = 0.956).

The length of the right coronal ulnar bowing in females was 212.75 ± 10.77 mm and the left was 210.71 ± 11.16 mm (P = 0.343), and the location of coronal bowing was 75.98 ± 18.84 mm in the right female ulna, and 76.01 ± 19.64 mm in the left (P = 0.992). Coronal bowing depth of the right female ulna was 7.86 ± 2.08 mm, and that of the left was 10.38 ± 1.77 mm (P = 0.854). In males, the length of right coronal ulnar bowing was 229.74 ± 10.83 mm, and that of the left was 229.05 ± 9.95 mm (P = 0.697). The coronal bowing location of the right male ulna was located at 79.24 ± 8.38 mm, and that of the left was 80.82 ± 8.28 mm (P = 0.268). The coronal bowing depth of the right male ulna was 9.34 ± 1.87 mm, and that of the left was 9.66 ± 1.79 mm (P = 0.306).

Ulnar bowing length in the sagittal plane was 217.56 ± 10.58 mm in the right and 214.99 ± 11.38 mm in the left (P = 0.236) among females. The location of sagittal bowing for females was 57.05 ± 29.82 mm in the right and 59.09 ± 29.20 mm in the left (P = 0.725). The depth of sagittal bowing for females was 6.33 ± 2.27 mm in the right and 6.72 ± 2.36 mm in the left (P = 0.401). In males, right sagittal bowing was located at 235.46 ± 11.48 mm and that of the left was 233.71± 10.81 mm (P = 0.361). Bowing location in the sagittal plane of males was located at 53.01 ± 35.29 mm for the right and 56.78 ± 38.15 mm for the left (P = 0.548). Sagittal depth was 7.03 ± 3.00 mm and 7.05 ± 3.09 mm on the right and left, respectively (P = 0.739) (Table 3).

**Table 3:** Twisting and bending measurement of the forearm bones.

			Female					Male				
			Right		Left		*P*	Right		Left		*P*
			Mean	SD	Mean	SD		Mean	SD	Mean	SD	
Radius	Coronal	Bow length (mm)	171.38	10.55	170.01	10.63	0.510	184.83	9.77	183.29	9.87	0.359
		Bow location (mm)	96.67	13.84	96.97	9.49	0.900	104.43	9.65	102.56	9.31	0.248
		Bow depth (mm)	10.45	2.08	10.38	1.77	0.854	11.95	1.85	12.15	1.90	0.533
	Sagittal	Bow length (mm)	171.92	10.39	170.92	10.45	0.623	183.87	10.76	182.53	9.44	0.439
		Bow location (mm)	111.67	23.08	104.81	22.26	0.126	119.02	25.96	118.91	24.52	0.980
		Bow depth (mm)	7.76	2.93	7.93	3.01	0.770	9.99	3.07	10.02	3.40	0.956
	Twisting (˚)		43.75	13.55	45.94	12.96	0.400	44.15	12.95	46.30	14.82	0.366
Ulna	Coronal	Bow length (mm)	212.75	10.77	210.71	11.16	0.343	229.74	10.83	229.05	9.95	0.697
		Bow location (mm)	75.98	18.04	76.01	19.64	0.992	79.24	8.38	80.82	8.28	0.268
		Bow depth (mm)	7.86	2.11	8.23	2.40	0.407	9.34	1.87	9.66	1.79	0.306
	Sagittal	Bow length (mm)	217.56	10.58	214.99	11.38	0.236	235.46	11.48	233.71	10.81	0.361
		Bow location (mm)	57.05	29.82	59.09	29.20	0.725	53.01	35.29	56.78	38.15	0.548
		Bow depth (mm)	6.33	2.27	6.72	2.36	0.401	7.03	3.00	7.05	3.09	0.976
	Twisting (˚)		22.29	13.45	17.82	12.72	0.084	16.41	12.01	15.72	12.30	0.739

#### C. Twisting

The twisting angle of the female radius was 43.75 ± 13.55˚ for the right, and the left was 45.94 ± 12.96˚ (P = 0.400). In the male radius, the twisting angle was 44.15 ± 12.95˚ for the right and 46.30 ± 14.82˚ for the left (P = 0.366).

The ulnar twisting angle of females was 22.29 ± 13.45˚ for the right and 17.82 ± 12.72˚ for the left (P = 0.084). Male ulnar twisting angle was 16.41 ± 12.01˚ for the right, and 15.72 ± 12.30 ˚ for the left (Table 3).

### Regional shape difference analysis of the radius and ulna

#### A. Radius

**Fig 5:**
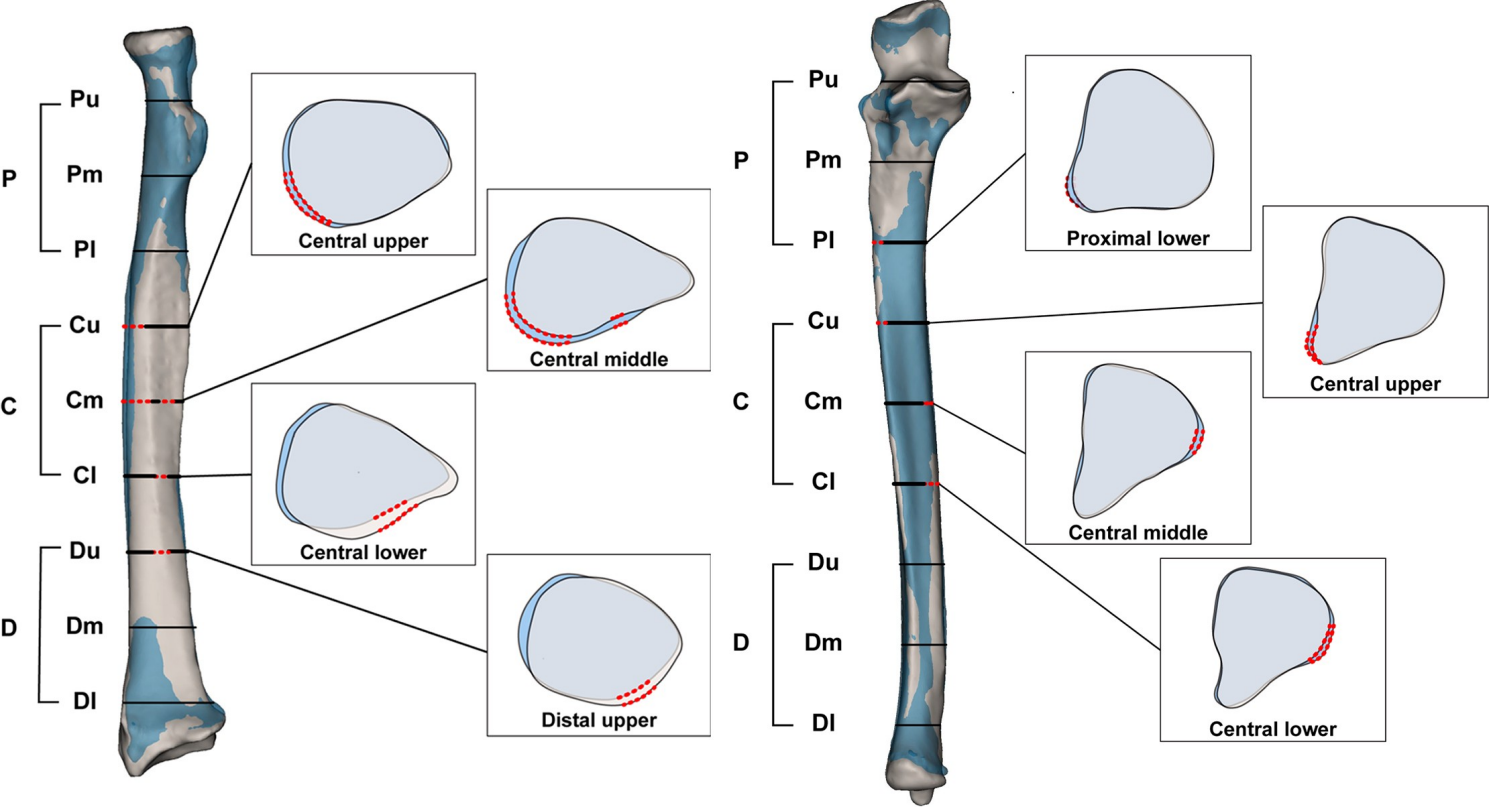
Regional shape difference of the radius and ulna. Angles with significant shape differences between bilateral bones are marked with dotted red lines on each cross-sectional image. **P**, proximal; **C**, central; **D**, distal; **Pu**, upper section of the proximal part; **Pm**, middle section of the proximal part; **Pl**, lower section of the proximal part; **Cu**, upper section of the central part, **Cm**, middle section of the central part, **Cl**, lower section of the central part, **Du**; upper section of the distal part, **Dm**; middle section of the distal part, **Dl**; lower section of the distal part.

1. Proximal
  Bilateral shape differences for the upper section of radii ranged from 0.01 mm to 3.18 mm. In the middle and lower sections of the proximal part, measurement values ranged from 0.00 mm to 2.77 mm for the middle section, and 0.00 mm to 3.27 mm for the lower section. Statistically significant shape differences between sides were not found in the upper to lower section of the proximal part (Fig 5).
2. Central
  Bilateral differences of the upper section of the central part ranged from 0.00 mm to 2.58 mm. There were significant differences from 210° to 260°, with the average difference being 0.56 mm (0.0 ≤ p ≤ 0.04). In the middle section of the central part, differences ranged from 0.00 mm to 3.07 mm. There were two independent regions with significant differences. Angle 140° to 150° exhibited 0.46 mm difference (0.03 ≤ p < 0.04). The mean difference for angle 200° to 260° was 0.49 mm (0.01 ≤ p < 0.05). In the lower section of the central part, differences between radii ranged from 0.01 mm to 3.23 mm. Angle 120° to 160° exhibited an average 0.50 mm gap (0.01 ≤ p ≤ 0.04).
3. Distal
  The upper section of the distal part exhibited differences ranging from 0.00 mm to 4.10 mm, with significant differences from angle 130° to 160°. The mean gap in that region was 0.49 mm (0.01 ≤ p ≤ 0.03). In the central and lower section of the distal part, differences ranged from 0.01 mm to 6.33 mm and 0.00 mm to 10.15 mm, but they were not significant.

#### B. Ulna

1. Proximal
  Differences for upper and central sections of proximal part of both ulnae ranged from 0.00 mm to 18.47 mm and 0.00 mm to 2.80 mm, respectively. The gaps in the lower section of the proximal part ranged from 0.01 mm to 3.21 mm and at 230° to 240°, the difference was significant at 0.58mm (0.02 ≤ p ≤ 0.04).
2. Central
  The upper to lower sections of the central part exhibited differences ranging from 0.00 mm to 3.80 mm. Significant differences were found in every section of the central ulna. These differences were present from 220° to 240° in the upper section of the central part (0.03 ≤ p < 0.05). From 90° to 110° in the middle section, and 80° to 140° in the lower section, the difference was 0.60 mm (0.00<p<0.05), and the averages of the differences were 0.55 mm, 0.53 mm, 0.60 mm, respectively.
3. Distal
  In the distal part of the ulna, differences ranged from 0.00 mm to 3.25 mm for the upper section. From 90° to 150° in the upper section, the difference was 0.48 mm (0.01≤p≤0.04). Differences in the central and lower sections of the distal part ranged from 0.00 mm to 0.27 mm and 0.00 mm to 0.41 mm, respectively, but the difference was not significant.

### Reliability of registration and alignment

Our results might be affected by factors related to registration and alignment. To assess the inter-rater reliability of our measures, we performed intraclass correlation coefficient (ICC) tests. First, we compared the lengths, twisting, and bowing of bilateral radii and ulnae and found that the ICCs were greater than 0.99 for both forearms. Then, we compared all measurement values of each section for both forearm bones and found that ICCs ranged from 0.98 to 0.99 for the radius and 0.82 to 0.99 for the ulna. All ICCs therefore satisfy criteria for reproducibility and data reliability.

## Discussion

The radius and ulna have a unique anatomical arrangement and complicated anatomical structure that enable the forearm bones to perform supination and pronation. Due to these distinct movements, complications after severe fracture such as posttraumatic malunion may occur. Despite that symptomatic malunion or pseudarthrosis can occur without symptoms or pain in some cases, posttraumatic malunion of the forearm bones needs to be addressed in many cases [21]. Malunited forearm bones can limit pronation and supination movement ranges, and result in unstable and painful DRUJ as well as cosmetic deformities. Because of these complications, forearm bone fractures must be restored to proper length and rotational alignment. However, corrections of comminuted forearm bone fractures are not simple. In this condition, applying CAOS and/or PSIs can have several potential advantages.

The most important precondition to utilize PSIs and CAOS is that the left and right bones must have similar features. Several researchers have described bilateral asymmetry of the forearm bones. Auerbach et al. examined bilateral measures of the upper and lower limb bones (the femur, tibia, humerus, and radius) [1]. Vroemen et al. described the bilateral symmetry of the radius and ulna through 3D analysis for planning corrective surgery of the distal radius and Gray et al. reported bilateral symmetry of the distal radii [22, 29]. These studies concentrated on only one bone or only on the distal parts of the forearm bones. Malunion can occur if bilateral forearm to the overall shape is not considered, and that leads to dysfunction of forearm bone movement [30, 31]. Furthermore, there is few information on regional asymmetry of the forearm bones.

In this study, we suggested differences in whole bone parameters and regional shape analysis of the forearm bones. Length, volume, bowing and twisting differences of the bilateral forearm bones were investigated first, then regional shape differences were analyzed. In particular, many researchers have reported that the radius and ulna have characteristic natural bowing and twisting [28, 32–36]. Several studies reported that deformities of the forearm bone diaphysis can cause 50% to 60% restriction of forearm bone rotation [31, 37]. According to Dumont et al., the maximum bilateral difference was over 30° [33]; therefore, bowing and twisting information may be essential for bone fracture reduction surgery. In our study, there were no statistically significant differences in bowing or twisting values between the two sides.

In the results of regional shape difference analysis, the proximal and distal sections of both forearms show large differences. The average bilateral difference of the proximal upper section of the radius was 1.23 mm (0.01 mm to 3.18 mm). In the distal lower section of the radius, the mean bilateral difference was 0.87 mm (0.00 mm to 10.15 mm). The average bilateral difference of the proximal upper section of the ulna was 0.99 mm, which was the largest bilateral difference. The minimum and maximum bilateral distances of the proximal upper section of the ulna were 0.99 mm to 18.47 mm, respectively. The mean bilateral difference of the distal lower section of the ulna was 0.58 mm (0.00 mm to 4.41 mm). The bilateral distance values at the ends of forearm bones had large standard deviations, but the differences were not significant. There was a large bilateral difference in the proximal ulna of 18.47 mm. This difference in the right and left ulna of this sample indicated a rapid change in the shape of the proximal articular portion, especially in the coronoid process. We observed the same phenomenon in other samples; there were seven of 132 samples with large gaps in the bilateral coronoid process, and this may be a characteristic feature of these samples. None of the samples had deformities associated with fractures or osteophytes that would exclude them from analysis. However, these samples did not show statistically significant differences between sides. Bilateral radii and ulnae were aligned and registered by the whole bone standard in this study. Whole bone registration can result in findings of differences in the ends of bones, because asymmetry of the left and right forearm bones frequently occurs in terms of length. Besides, proximal and distal articulations of the radius and ulna have complex shapes. For these reasons, large bilateral differences were detected in the proximal and distal ends of both bones. Bilateral forearm bones should be aligned and registered by proximal or distal ends in studies of asymmetry of articulation shape.

On the anterior surface of the radius, significant shape differences were found in the upper section of the central part to the upper section of the distal part. On the anterior surface of the ulna, proximal lower to central lower sections show significant shape differences (Fig 1). These sections of both forearm bones are equivalent to the diaphysis. In contrast to the anterior surface, we did not observe significant differences of the posterior surface. We hypothesize that these results are related to muscle origins. The origin of the flexor pollicis longus muscle is located on the mid-anterior surface of the radius, and the origin of the flexor digitorum profundus muscle is located on the proximal two thirds of the anterior surface of the ulna. Unilateral side muscles can be developed more than the contralateral side, because most people use their dominant hand more than their less dominant hand. Asymmetrical development of the forearm muscles might result in asymmetrical development of the forearm bones. We did not observe asymmetry of the posterior surfaces of the forearm bones. Many muscles, such as the extensor carpi ulnaris, abductor pollicis longus, extensor pollicis longus, extensor pollicis brevis, and extensor indicis are located in the posterior compartment; thus, each muscle has a smaller origin than do the muscles of the anterior compartment. Furthermore, the origins of the posterior compartment muscles are mainly located on the interosseous membrane rather than the bone surface.

Our study has several limitations. First, we do not have handedness information for the cadavers examined in this study. According to a study by Sander et al., left-handed subjects have higher and larger left elbows than right-handed subjects [38]. A second limitation of our study regards the alignment of the 3D models of forearm bones. We aligned all 3D models using certain standards, but the procedures were performed manually. We randomly selected 14 samples and performed ICC tests to assess the reliability of this method. Because the ICCs ranged from 0.82 to 0.98, we considered our alignment method to be reliable. The final limitation of our study is that the CT data utilized for this research was derived entirely from South Korean adults and may not be generalized to other regional or demographic samples. Nevertheless, our results may be helpful for PSIs development and CAOS, because we analyzed a large sample of 3D models. A third limitation is that this study sought to investigate bilateral shape differences using global registration to examine entire bones. The results have shown larger differences in the proximal and distal ends of both forearm bones; however, these differences were not statistically significant. Registration was performed for the entire bone model in this study, which may not be adequate for designing PSIs and/or CAOS of particular proximal and/or distal areas. Further research on asymmetry of the proximal and distal end of the forearm bone is needed.

Our findings suggest there is are no statistically significant differences in whole bone parameters including length, volume, bowing or twisting in the bilateral forearm bones. In regional shape analysis, that was little asymmetry between bilateral forearm bones. Asymmetries occurred primarily on the anterior surface of the radius and ulna diaphysis. However, the average bilateral differences between the forearm bones were small, around 0.5 mm. Utilizing contralateral side forearm bones for PSIs development and CAOS of the affected sides may be feasible.

## Supporting information

S1 File Measurement results of all parameters. This file contains length, volume, torsion, bending, and regional shape differences data.
(XLSX)
